# Optimizing correction and restoring alignment: Outcomes of bone-substitute augmented short-segment fixation in thoracolumbar burst fractures, a descriptive series from Pakistan

**DOI:** 10.1097/MD.0000000000050300

**Published:** 2026-08-21

**Authors:** Abdullah Ayish, Muhammad Faisal Ejaz, Abdul Haseeb Butt, Zehra Fatima, Waleed Tariq, Muhammad Momin Khan, Muhammad Idrees, Ajay Pandeya

**Affiliations:** a Department of Orthopedic and Spine Surgery, Lahore General Hospital, Lahore, Punjab, Pakistan; b Department of Internal Medicine, Isra University, Sindh, Pakistan; c Department of Thoracic Surgery, Mayo Hospital, Lahore, Punjab, Pakistan; d Department of Internal Medicine, Liaquat University Hospital, Hyderabad, Pakistan; e Department of Internal Medicine, Lahore General Hospital, Lahore, Punjab, Pakistan; f Department of Medicine, KIST Medical College, Lalitpur, Bagmati, Nepal.

**Keywords:** case series, kyphotic angle, short-segment fixation, Thoracolumbar burst fracture, vertebral height

## Abstract

Thoracolumbar burst fractures are a major cause of spinal morbidity. While long-segment posterior fixation is traditionally used, short-segment fixation augmented with bone substitutes has been proposed to preserve motion segments while maintaining stability. This study aimed to evaluate radiological outcomes, specifically kyphotic angle correction and vertebral body height restoration, following short-segment fixation with bone substitute in patients with unstable thoracolumbar burst fractures. This retrospective descriptive case series was conducted at a tertiary care center in Sindh, Pakistan, from July 2022 to January 2023. Patients aged 16 to 65 years with single-level unstable thoracolumbar burst fractures (Arbeitsgemeinschaft für Osteosynthesefragen type A3/A4) undergoing short-segment posterior fixation with calcium hydroxyapatite augmentation were included. Kyphotic angle and vertebral body height were measured at baseline and at 3 months post-op using standardized methods. Statistical comparisons were performed using paired *t*-tests, with *P* < .05 considered significant. A total of 82 patients were analyzed, including 47 males (57.3%) and 35 females (42.7%), with a mean age of 48.4 ± 8.2 years. Fractures were classified as A3 (53.7%) and A4 (46.3%). Mean vertebral body height increased from 48.7 ± 1.2 mm preoperatively to 86.7 ± 3.2 mm postoperatively (*P* < .001). Mean kyphotic angle decreased from 20.4 ± 2.3° to 6.7 ± 2.1° (*P* < .001). Short-segment posterior fixation with bone substitute was associated with improved vertebral body height and reduction in kyphotic deformity in patients with unstable thoracolumbar burst fractures. Given the descriptive design and absence of a control group, these findings should be interpreted as observational. Further comparative studies with long-term follow-up are required to establish durability and clinical effectiveness.

**Highlights**

- Short segment posterior fixation augmented with bone substitute effectively restored vertebral height and corrected kyphosis across all patient subgroups.
- There was no significant difference in radiological outcomes among the subgroups analyzed.
- Multicenter studies with long term follow up are needed to assess durability, failure rates, and optimal patient selection.

## 1. Introduction

Thoracolumbar burst fractures, accounting for 20 to 60% of spinal fractures, typically result from high-energy axial loading forces.^[1,2]^ Such fractures disrupt the anterior and middle spinal columns, potentially resulting in loss of spinal stability and the development of neurological impairment.^[3]^ Surgical intervention is often indicated in cases of significant neurological impairment or severe deformity.^[1,4]^ Nevertheless, there remains considerable debate among spine surgeons regarding which surgical route – posterior, anterior, or a combination of both – offers the best outcomes.

The posterior approach remains the most employed method for thoracolumbar burst fractures.^[4–6]^ This technique permits indirect decompression via ligamentotaxis and provides spinal stabilization without requiring anterior access. In contrast, the anterior approach allows for direct decompression and fixation but typically demands a more invasive exposure, with a higher risk of complications.^[5]^

Recent research has investigated different strategies to improve the effectiveness of short-segment posterior fixation. Approaches such as using bone graft substitutes or reinforcing the fractured vertebra with extra screws have demonstrated potential in better restoring vertebral height and correcting kyphotic deformity.^[7,8]^ A clinical study by Liao et al demonstrated that short-segment fixation with bone augmentation resulted in a postoperative kyphotic angle of 6.8 ± 4.6° and vertebral body height of 86.3 ± 10.9%, indicating effective deformity correction and spinal stability.^[8]^

Despite these developments, consensus on the best surgical approach has not been reached. While some surgeons favor conventional long-segment fixation to maximize stability, others support short-segment fixation augmented with bone substitutes as a way to maintain spinal mobility.^[9–11]^ The purpose of this study is to assess radiographic results, focusing on the average postoperative kyphotic angle and vertebral body height in patients with unstable thoracolumbar burst fractures treated with short-segment fixation using bone substitutes. Furthermore, the study aims to provide regional data to help fill a gap in the existing literature on this subject.

## 2. Methodology

### 2.1. Study settings and design

This retrospective descriptive case series was conducted int the Department of Orthopedic & Spine Surgery, South City Hospital, Sindh, from July 11, 2022, to January 11, 2023.This case series has been reported in line with the Strengthening the Reporting of Observational Studies in Epidemiology Guideline.^[12]^

### 2.2. Inclusion criteria

Patients aged 16 to 65 years with single-level thoracolumbar burst fractures classified as Arbeitsgemeinschaft für Osteosynthesefragen (AO) Spine type A3 or A4, who were classified as American Society of Anesthesiologists (ASA) physical status I or II and underwent surgical treatment within one week of injury, were included.

### 2.3. Exclusion criteria

Patients were excluded if they had local or systemic infection, a history of heart failure (pro-B-type natriuretic peptide > 125 pg/mL), severe renal impairment (estimated glomerular filtration rate < 15 mL/min), liver cirrhosis, bleeding disorders, or were currently receiving anticoagulant therapy.

### 2.4. Sample size and sampling technique

A total of 82 patients were included using non-probability consecutive sampling. The sample size was calculated using a 95% confidence level, with an absolute precision of 0.10, based on an expected postoperative kyphotic angle of 6.8 ± 4.6.

### 2.5. Ethical considerations

Ethical approval was obtained from the Institutional Review Board of South City Hospital, Sindh, Pakistan (Protocol No. 122312; approval date: July 10, 2022). The study was conducted in accordance with the Declaration of Helsinki (2013 revision). Written informed consent was obtained from all participants before inclusion. Participants were informed about the study objectives, procedures, risks, and benefits, including consent for the use of anonymized clinical and radiological data.

### 2.6. Surgical technique and radiological assessment

All patients underwent short-segment posterior fixation under general anesthesia using a standardized surgical approach performed by an experienced spine surgeon. Patients were positioned prone, and a posterior midline approach was used to expose the affected spinal segment. Monoaxial pedicle screws were inserted into the fractured vertebra and the adjacent vertebrae. Fracture reduction and indirect decompression were achieved via ligamentotaxis, followed by stabilization with internal fixation reinforced using cross-links. The fractured vertebral body was then augmented with a calcium hydroxyapatite bone substitute (standard medical-grade synthetic bone graft substitute), with an approximate volume of 2 to 4 mL applied per vertebra in a standardized manner across all cases. Minor variations in technique were limited to patient-specific anatomical considerations; however, the overall procedural steps were consistent throughout the cohort.

Radiological evaluation was performed using X-ray, computed tomography, and magnetic resonance imaging. The kyphotic angle was measured using the Cobb method between the superior endplate of the vertebra above and the inferior endplate of the vertebra below the fracture level. Vertebral body height was assessed by measuring the anterior vertebral height at the fractured level.

### 2.7. Outcomes

The primary outcomes were: Change in kyphotic angle, and Restoration of vertebral body height.

Secondary outcomes included subgroup comparisons across: age, gender, weight, ASA class, and fracture type.

Radiological measurements were recorded: Preoperatively, and postoperatively at 3 months.

### 2.8. Data collection and statistical analysis

Data were analyzed using Statistical Package for the Social Sciences version 23.0. Continuous variables were expressed as mean ± standard deviation, while categorical variables were presented as frequencies and percentages. The distribution of continuous variables was assessed for normality using the Shapiro–Wilk test, and parametric assumptions were verified before analysis. Preoperative and postoperative radiological parameters, including vertebral height and kyphotic angle, were compared using paired *t*-tests. For subgroup comparisons based on age, gender, weight, ASA classification, and fracture type, independent t-tests or one-way analysis of variance (ANOVA) were applied as appropriate. A *P*-value of <.05 was considered statistically significant.

## 3. Results

### 3.1. Demographics of participants

**Figure 1.**
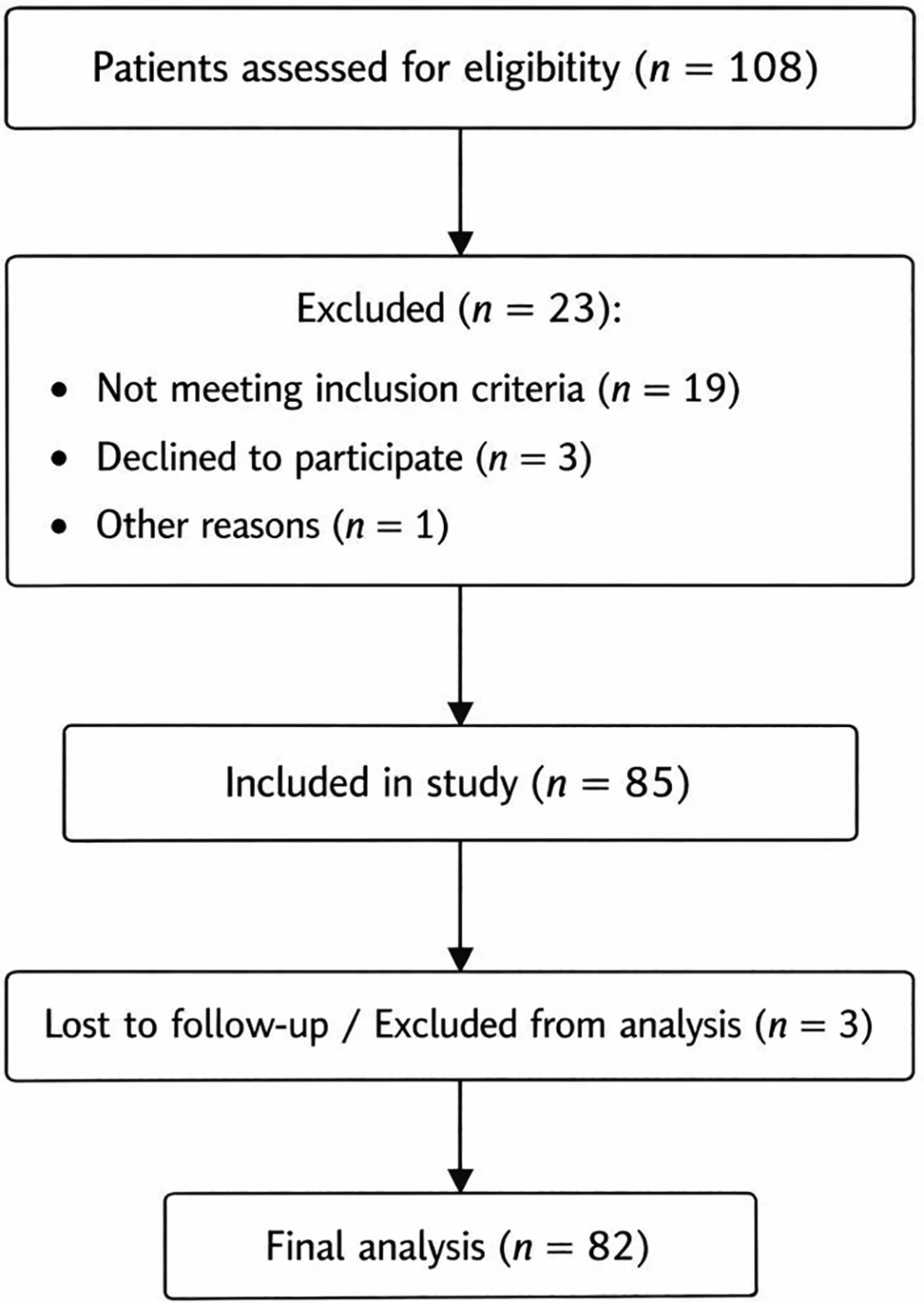
STROBE flow diagram showing patient selection, exclusions, and final analysis (N = 82). STROBE = Strengthening the Reporting of Observational Studies in Epidemiology.

A total of 82 patients were included in the final analysis (Fig. 1). The mean age was 48.43 ± 8.17 years. Most patients were in the 41 to 65 years age group (87.8%, n = 72), while 12.2% (n = 10) were between 16 to 40 years. The mean body weight was 72.35 ± 13.77 kg (Table 1).

**Table 1:** Demographic and clinical characteristics of patients (N = 82).

Characteristic	Category	n (%) or Mean ± SD
Age (yr)	Mean ± SD	48.4 ± 8.2
	16–40	10 (12.2%)
	41–65	72 (87.8%)
Weight (kg)	Mean ± SD	72.35 ± 13.77
Gender	Male	47 (57.3%)
	Female	35 (42.7%)
ASA classification	I	36 (43.9%)
	II	46 (56.1%)
Fracture type	A3	44 (53.7%)
	A4	38 (46.3%)

ASA = American Society of Anesthesiologists, SD = standard deviation.

Regarding gender distribution, 57.3% (n = 47) were male and 42.7% (n = 35) were female. In terms of ASA classification, 43.9% (n = 36) were ASA I, while 56.1% (n = 46) were ASA II.

Fracture classification observed that 53.7% (n = 44) were AO type A3 fractures, while 46.3% (n = 38) were type A4 fractures.

### 3.2. Radiological outcomes

Radiological parameters observed improvement following surgical intervention. The mean vertebral body height increased from 48.7 ± 1.2 mm preoperatively to 86.7 ± 3.2 mm postoperatively. The mean increase was 38.0 mm (95% confidence interval: 36.9–39.1; *P* < .001, paired *t*-test).

Similarly, the mean kyphotic angle decreased from 20.4 ± 2.3° preoperatively to 6.7 ± 2.1° postoperatively, representing a mean reduction of 13.7° (95% confidence interval: 12.9–14.5; *P* < .001, paired *t*-test).

### 3.3. Stratification analysis

Postoperative vertebral height and kyphotic angle were further analyzed across subgroups based on age, gender, weight, ASA class, and fracture type using independent t-tests or one-way ANOVA as appropriate (Table 2).

**Table 2:** Stratification of post-operative vertebral height and kyphotic angle (N = 82).

Variable	Subgroup	N	Vertebral height (cm) mean ± SD	Kyphotic angle (°) mean ± SD	*P*-value (height/angle)
Age	16–40	10	86.60 ± 0.15	6.68 ± 0.13	.393/ .423
	41–65	72	86.69 ± 0.34	6.71 ± 0.11	–
Gender	Male	47	86.63 ± 0.16	6.71 ± 0.11	.092/ .762
	Female	35	86.75 ± 0.46	6.70 ± 0.11	–
Weight	40–60 kg	29	86.62 ± 0.15	6.68 ± 0.10	.244/ .218
	>60 kg	53	86.71 ± 0.39	6.72 ± 0.11	–
ASA	I	36	86.69 ± 0.39	6.71 ± 0.11	.779/ .515
	II	46	86.75 ± 0.27	6.70 ± 0.11	–
Fracture type	A3	44	86.68 ± 0.38	6.69 ± 0.11	.970/ .416
	A4	38	86.68 ± 0.25	6.71 ± 0.12	–

ASA = American Society of Anesthesiologists, SD = standard deviation.

No statistically significant differences were observed in postoperative vertebral height or kyphotic angle across these subgroups (all *P* > .05).

Post-operative vertebral height ranged from 86.60 to 86.75 cm, and the mean postoperative kyphotic angle ranged from 6.68 to 6.71° across all subgroups.

## 4. Discussion

This study evaluated 82 patients with unstable thoracolumbar burst fractures who underwent short-segment posterior fixation augmented with calcium hydroxyapatite. The cohort had a mean age of 48 years and included slightly more males than females. Postoperative assessment showed a marked improvement in vertebral height and a significant reduction in kyphotic angles compared to preoperative values. These findings were observed in the study population and reflect radiological changes following the intervention. However, as a retrospective descriptive case series, the findings should be interpreted as observational and hypothesis-generating rather than definitive.

These findings align with increasing evidence supporting the use of augmented short-segment fixation methods. A recent meta-analysis on thoracolumbar burst fractures reported that adding intermediate screws or other augmentation techniques led to better radiographic outcomes, including improved kyphosis correction and vertebral height restoration, although overall clinical outcomes were comparable primarily to conventional short-segment constructs.^[13]^ Zhang and colleagues, through a meta-analysis, showed that combining vertebroplasty or fracture-level augmentation with short-segment fixation significantly enhanced Cobb angle correction and decreased anterior vertebral height loss compared to non-augmented techniques, without leading to longer operative times or increased blood loss.^[14]^ In biomechanical studies, segmental fixation, including pedicle screws at the fractured vertebra, offers greater stability and reduced stress on adjacent screws versus non-segmental constructs.^[15–17]^

Comparisons of short-versus long-segment fixation provide valuable insights. Aly et al, in a meta-analysis, reported no significant differences between the 2 approaches in terms of radiographic results, functional recovery, neurological improvement, or rates of implant failure, suggesting that longer constructs may not be necessary in all cases.^[18]^ However, a caveat arises: long-segment fixation may better prevent late loss of correction in select high-risk cases.^[19]^ For example, a recent study comparing long- and short-segment fixation found that long-segment constructs provided slightly better preservation of the Cobb angle at final follow-up (mean difference approximately 2.5°) and reduced implant failure, though this was accompanied by longer operative times and increased blood loss.^[20]^ Additionally, a systematic review examining factors linked to failure in posterior short-segment instrumentation reported an overall failure rate of around 14%, with higher Load Sharing Classification scores, increased preoperative kyphosis, and more severe vertebral compression identified as significant risk factors.^[21]^ This suggests that both approaches may achieve comparable outcomes in selected patient populations, although direct causal comparisons cannot be drawn from this study.

The improvements we observed in kyphotic correction and vertebral height are consistent with previously published data. For instance, Sanderson et al reported a 14% implant failure rate and similar radiographic and clinical outcomes using short-segment fixation without fusion.^[22]^ The long-term follow-up study by Kultur et al showed that short-segment posterior instrumentation (with fusion) maintained vertebral body height and wedge angle correction over 20 years, with minimal later deterioration.^[23]^ These observations suggest that achieving and maintaining an adequate initial correction can provide long-term stability even with limited fixation. Similarly, Luo et al’s review on short-segment fixation with transpedicular bone grafting highlights that shorter constructs can decrease operative time and blood loss, preserve spinal motion segments, and, when augmented, help prevent loss of correction.^[24]^

### 4.1. Strengths and limitations

A significant strength of this study is its relatively large cohort (n = 82) for a single-center surgical series, combined with a standardized surgical approach (short-segment fixation with bone substitute) and consistent radiographic assessment. Stratified analyses suggest that outcomes were similar across different patient subgroups; however, these findings should be interpreted cautiously due to the observational design and limited sample size. Moreover, the findings contribute valuable regional data, particularly in contexts where resource limitations may restrict the use of more extensive fixation techniques.

Nevertheless, several limitations should be considered. First, as a descriptive case series without a randomized or matched control group (e.g., long-segment fixation or short-segment fixation without augmentation), the study cannot establish causal relationships. Second, the lack of multivariable analysis means that potential confounding factors such as fracture severity, bone quality, and patient characteristics were not adjusted for, which may influence the observed outcomes. Third, the follow-up period focuses on early postoperative outcomes, preventing evaluation of long-term correction loss, implant failure, or functional measures such as pain and quality of life. Fourth, the absence of load-sharing scores or detailed imaging predictors limits the ability to analyze risk factors for failure. Finally, as a single-center study, the findings may have limited applicability to other populations or surgical settings.

## 5. Conclusion

This study supports that short-segment posterior fixation augmented with a bone substitute is associated with improvement in vertebral height and kyphotic alignment in this cohort. The observed radiological changes appeared similar across different patient subgroups; however, these findings should be interpreted cautiously given the study’s observational design and limited sample size.

Given the absence of a control group and long-term follow-up and comparative controls, conclusions regarding comparative effectiveness and durability cannot be made. Further prospective, multicenter studies with longer follow-up and appropriate comparative groups are needed to better evaluate outcomes, assess implant durability, and refine patient selection.

## Author contributions

**Conceptualization:** Abdullah Ayish, Muhammad Faisal Ejaz, Abdul Haseeb Butt, Zehra Fatima, Waleed Tariq, Muhammad Momin Khan, Muhammad Idrees, Ajay Pandeya.

**Data curation:** Abdullah Ayish, Muhammad Faisal Ejaz, Abdul Haseeb Butt, Zehra Fatima, Waleed Tariq, Muhammad Momin Khan.

**Methodology:** Abdullah Ayish, Muhammad Faisal Ejaz, Abdul Haseeb Butt, Muhammad Idrees.

**Supervision:** Abdullah Ayish.

**Writing – original draft:** Abdullah Ayish, Muhammad Faisal Ejaz, Abdul Haseeb Butt, Zehra Fatima, Waleed Tariq, Muhammad Momin Khan, Muhammad Idrees, Ajay Pandeya.

**Writing – review & editing:** Ajay Pandeya.
